# Urgent Endovascular Treatment of a Pancreatitis-Related Pseudoaneurysm Identified on Follow-Up CT

**DOI:** 10.3390/diagnostics16213702

**Published:** 2026-11-09

**Authors:** Vladimir Cvetic, Sonja Lukendic, Milica Mitrovic, Aleksandra Jankovic, Borivoje Lukic, Aleksandar Bogdanovic, Predrag Zdujic, Dejan Radenkovic, Jelena Djokic Kovac

**Affiliations:** 1 Faculty of Medicine, University of Belgrade, Dr Subotica No. 8, 11000 Belgrade, Serbia; drvladimircvetic@gmail.com (V.C.);; 2 Center for Radiology and Magnetic Resonance Imaging, University Clinical Centre of Serbia, Pasterova No. 2, 11000 Belgrade, Serbia; 3 Clinic for Digestive Surgery, University Clinical Centre of Serbia, Koste Todorovica Street, No. 6, 11000 Belgrade, Serbia

**Keywords:** chronic pancreatitis, pseudoaneurysm, hepatic artery, endovascular therapy, covered stent

## Abstract

Visceral artery pseudoaneurysm (VA-PSA) is an uncommon but life-threatening complication of acute-on-chronic pancreatitis, particularly in the presence of walled-off necrosis (WON). Early radiological recognition is essential because of the high risk of rupture. We present the case of a 51-year-old man with chronic pancreatitis who developed a pseudoaneurysm arising from the proper hepatic artery within a pancreatic head necrotic collection. Follow-up contrast-enhanced computed tomography (CT) demonstrated a new arterial-phase enhancing focus within the WON. Digital subtraction angiography (DSA) confirmed the pseudoaneurysm, which was successfully excluded with a covered stent placed via a right transfemoral approach. Post-procedural recovery was uneventful, and short-term follow-up CT showed persistent exclusion of the lesion.

The patient, a 51-year-old man, had a long-standing history of recurrent alcohol-related pancreatitis complicated by endocrine pancreatic insufficiency with resulting diabetes mellitus, he also had arterial hypertension. During the initial hospitalization at the referring regional hospital, contrast-enhanced CT showed acute-on-chronic pancreatitis with an acute necrotic collection in the region of the pancreatic head, without evidence of a pseudoaneurysm (Figure 1 and Figure 2). He was admitted with persistent abdominal pain, loss of appetite and weight loss related to chronic pancreatitis, and a three-month follow-up contrast-enhanced CT was performed during this hospitalization (Figure 3). On admission, he was hemodynamically stable and normotensive, with no clinical signs of acute gastrointestinal or intra-abdominal bleeding.

Laboratory tests showed a normal leukocyte count, mildly elevated inflammatory markers (C-reactive protein 16.3 mg/L) and mild anemia (hemoglobin 108 g/L, hematocrit 0.32). Serum pancreatic enzymes were slightly elevated (amylase 125 U/L, lipase 115 U/L). Liver biochemical tests showed a cholestatic pattern, with markedly elevated alkaline phosphatase (1255 U/L) and gamma-glutamyl transferase (2463 U/L) and moderately elevated transaminases (AST 124 U/L, ALT 72 U/L). The cholestatic pattern was most likely attributable to post-inflammatory stenosis of the distal common bile duct in the setting of chronic pancreatitis and to compression by the pancreatic head collection, as CT showed dilatation of the common bile duct (up to 10 mm) with wall thickening suggestive of cholangitis; chronic alcohol-related liver injury may also have contributed. These abnormalities were already present on admission, before the endovascular procedure.

On admission, the patient’s clinical and laboratory profile did not suggest a severe systemic inflammatory response: hemodynamic stability, absence of hemoconcentration, a normal leukocyte count, only mildly elevated C-reactive protein, age under 60 years and absence of pleural effusion on CT are all features associated with a favorable prognosis in acute pancreatitis [1]. Nevertheless, the development of a necrotic collection placed the index episode in at least the moderately severe category according to the revised Atlanta classification [2].

Visceral artery pseudoaneurysm is a rare but potentially fatal complication of chronic pancreatitis because of its propensity to rupture and cause massive hemorrhage. Mortality approaches 90% when hemorrhage is left untreated, whereas after definitive endovascular or surgical treatment, the reported mortality ranges from approximately 15% to 50%, depending on the patient’s clinical condition, lesion location and treatment modality [3,4]. When a pancreatitis-related pseudoaneurysm ruptures into the pancreatic duct, it may cause hemosuccus pancreaticus, a rare cause of gastrointestinal bleeding characterized by intermittent passage of blood through the major duodenal papilla; peripancreatic pseudoaneurysm complicating chronic pancreatitis is its most common underlying cause [5]. Therefore, follow-up of patients with chronic pancreatitis, combining clinical assessment with imaging tailored to the clinical course, may facilitate early detection of vascular complications and timely selection of therapeutic strategies [3,4]. The mechanism of pseudoaneurysm formation involves persistent pancreatic inflammation and proteolytic enzyme activity, which may weaken adjacent arterial walls or lead to vascular erosion in the setting of pseudocyst formation and local tissue necrosis [6]. In addition, inflammation-driven phenotypic switching of vascular smooth muscle cells, which has been implicated in arterial wall weakening in other vascular diseases [7,8], may also contribute, although this has not been demonstrated in pancreatitis-related pseudoaneurysms.

The reported prevalence of pseudoaneurysms in patients with chronic pancreatitis ranges from approximately 4% to 10% [9]. The splenic artery is the most frequently involved (60–65%), followed by the gastroduodenal (20–25%), pancreaticoduodenal (10–15%), hepatic (5–10%) and left gastric arteries (2–5%) [10,11]. Importantly, the true incidence may be underestimated, as contrast-enhanced imaging is not universally performed in asymptomatic patients, potentially allowing clinically silent vascular lesions to remain undetected. In a literature review by Jia et al., only 12 cases of hepatic artery pseudoaneurysm secondary to chronic pancreatitis were identified in the English-language literature, most of which involved the common hepatic artery and only one involved the proper hepatic artery [12]. The location in the proper hepatic artery in our patient is therefore exceptional.

Considering the size (approximately 48 mm) and anatomical location of the pseudoaneurysm within the necrotic collection (Figure 4), the associated risk of rupture and the patient’s clinical condition, we opted for endovascular treatment.

The pseudoaneurysm arose from the proper hepatic artery through a narrow neck measuring approximately 1.3 mm. Through a right transfemoral approach using a 6 Fr introducer sheath, the celiac trunk and the common hepatic artery were selectively catheterized. The parent artery measured 5 mm in diameter at both the proximal and distal landing zones, and a 6 × 38 mm balloon-expandable BeGraft Peripheral covered stent (Bentley InnoMed GmbH, Hechingen, Germany) was deployed across the pseudoaneurysm neck, providing approximately 20% oversizing and a secure seal (Figure 5). Selective angiography of the superior mesenteric artery showed no retrograde filling of the excluded pseudoaneurysm sac (Figure 6). Hemostasis at the puncture site was achieved by manual compression, without complications.

Unfractionated heparin (5000 IU) was administered intravenously during the procedure. After stent deployment, dual antiplatelet therapy with acetylsalicylic acid 100 mg and clopidogrel 75 mg daily was started to prevent in-stent thrombosis, after weighing this benefit against the bleeding risk associated with peripancreatic inflammation; pantoprazole 20 mg was added twice daily for gastroprotection. The patient was discharged on dual antiplatelet therapy for 3 months, which was to be followed by single antiplatelet therapy; the concomitant portal vein thrombosis was managed conservatively in consultation with the surgical and gastroenterology teams. The patient recovered well and was discharged home in a stable condition five days after the intervention. Follow-up contrast-enhanced CT performed three months later demonstrated persistent exclusion of the pseudoaneurysm, with preserved stent patency and no evidence of recurrence, other vascular complications or procedure-related abnormalities (Figure 7). Given the relatively short follow-up, continued surveillance will be essential to establish the long-term durability of the treatment.

Splanchnic vein thrombosis is a recognized local complication of pancreatitis, with a pooled prevalence of approximately 11.6% in chronic pancreatitis; it is attributed to peripancreatic inflammation with endothelial injury, compression by adjacent collections and inflammation-induced activation of coagulation. The portal vein is involved in approximately 3.5% of these patients [13]. In our patient, the acute necrotic collection extending into the hepatoduodenal ligament (Figure 1) was its most likely cause. The thrombosis subsequently evolved into chronic occlusion of the main portal vein with cavernous transformation, portosystemic collaterals and signs of secondary portal hypertension, all of which persisted on follow-up CT.

Visceral artery pseudoaneurysm is a rare but serious vascular complication of acute-on-chronic pancreatitis. More common complications of chronic pancreatitis include biliary obstruction with jaundice, duodenal stenosis due to inflammatory or fibrotic changes, splenic vein thrombosis and pseudocyst formation, whereas pseudoaneurysms occur considerably less often but carry substantial clinical risk [14].

In patients with chronic pancreatitis, particularly in the presence of large peripancreatic fluid collections or walled-off necrosis, delayed vascular complications should be considered during follow-up. Large, well-demarcated peripancreatic collections exceeding 5 cm have been associated with a higher rate of complications [15], and pancreatic necrosis is associated specifically with an increased risk of major hemorrhage [16]; however, this cut-off should not be regarded as a universally established threshold. When such complications are suspected, contrast-enhanced multiphasic CT angiography enables reliable detection of pseudoaneurysm formation and other hemorrhagic complications, while Doppler ultrasound may serve as a useful noninvasive adjunct for interval assessment. As evidence on the optimal timing of follow-up imaging remains limited, imaging strategies should be individualized according to the clinical course, symptoms and the presence of specific local complications, with prompt re-imaging if new symptoms develop [16,17]. Hepatic artery pseudoaneurysms should be repaired as soon as they are diagnosed, irrespective of size [18].

Management of pancreatitis-related visceral artery pseudoaneurysms should be individualized according to the patient’s hemodynamic status, vascular anatomy and available therapeutic options. Surgery is reserved for hemodynamically unstable patients and for cases of failed endovascular treatment, as operative management in chronic pancreatitis is technically demanding and carries increased risk. In contrast, for clinically stable patients with access to interventional radiology, minimally invasive endovascular treatment is the preferred first-line approach [4]. Digital subtraction angiography enables accurate localization of the bleeding vessel and allows immediate therapeutic intervention. Transcatheter embolization remains the most widely used technique, with high technical success rates in achieving rapid hemostasis [4,9]. Coils or microcoils are the most commonly used embolic agents, whereas liquid embolic agents (glue) are reserved for selected cases, such as marked vessel tortuosity or recurrence after coiling, because of the higher risk of non-target embolization [11]. Thrombin may be injected either through a catheter or by direct percutaneous or endoscopic ultrasound-guided puncture of the sac, whereas gelatin sponge (Gelfoam) and vascular plugs may be used in combination with coils [11]. However, in lesions of major visceral arteries, where preservation of arterial perfusion is crucial, covered stent implantation is an effective alternative that excludes the pseudoaneurysm while maintaining distal blood flow [19]. When a pseudoaneurysm arises from a major visceral artery such as the proper hepatic artery, coil embolization or parent vessel occlusion carries a non-negligible risk of downstream ischemic complications, including hepatic necrosis or biliary ischemia. In contrast to the case reported by Jia et al., in which a ruptured pseudoaneurysm of a small distal branch of the right hepatic artery was treated by glue embolization [12], the proximal location of the lesion in our patient, together with the coexisting portal vein thrombosis, made preservation of hepatic arterial inflow essential, favoring covered stent placement over embolization. Furthermore, the absence of the pseudoaneurysm on the initial CT and its detection on follow-up CT within three months underscore the importance of imaging surveillance in patients with large walled-off necrotic collections. In our clinical practice, both selective embolization and covered stent placement are routinely used, depending on lesion morphology and the need to preserve arterial patency. Early diagnosis followed by prompt endovascular management may reduce the need for emergency surgery and has been associated with favorable clinical outcomes.

## Figures and Tables

**Figure 1:**
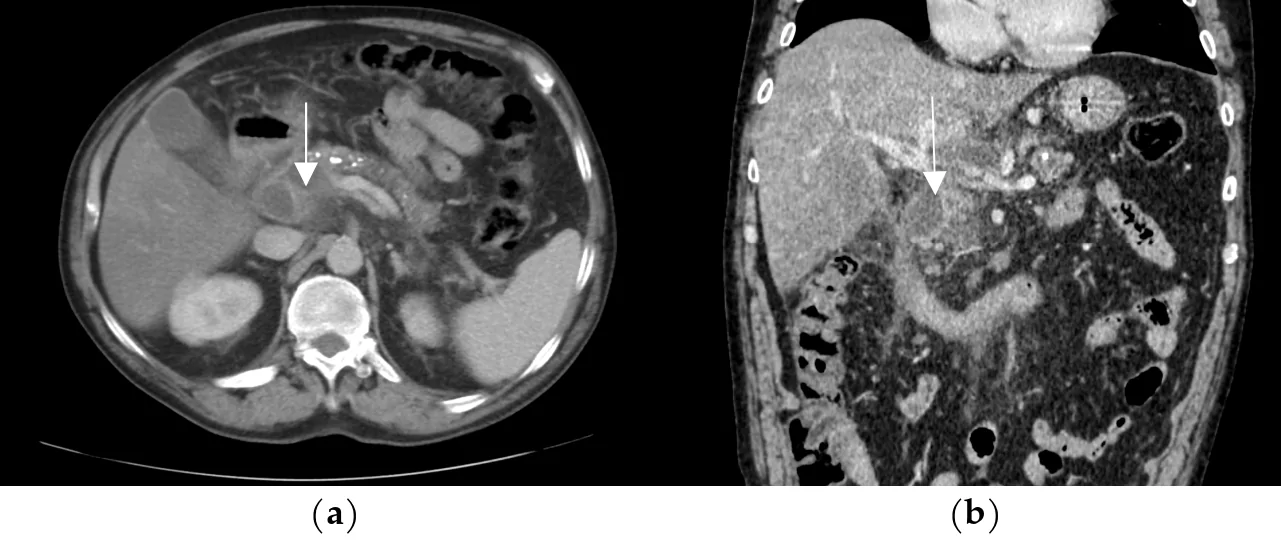
Contrast-enhanced CT performed during the initial hospitalization at the referring regional hospital shows signs of acute-on-chronic pancreatitis with an acute necrotic collection in the region of the pancreatic head and the hepatoduodenal ligament (white arrow) (**a**). Portal vein thrombosis is also seen (white arrow) (**b**).

**Figure 2:**
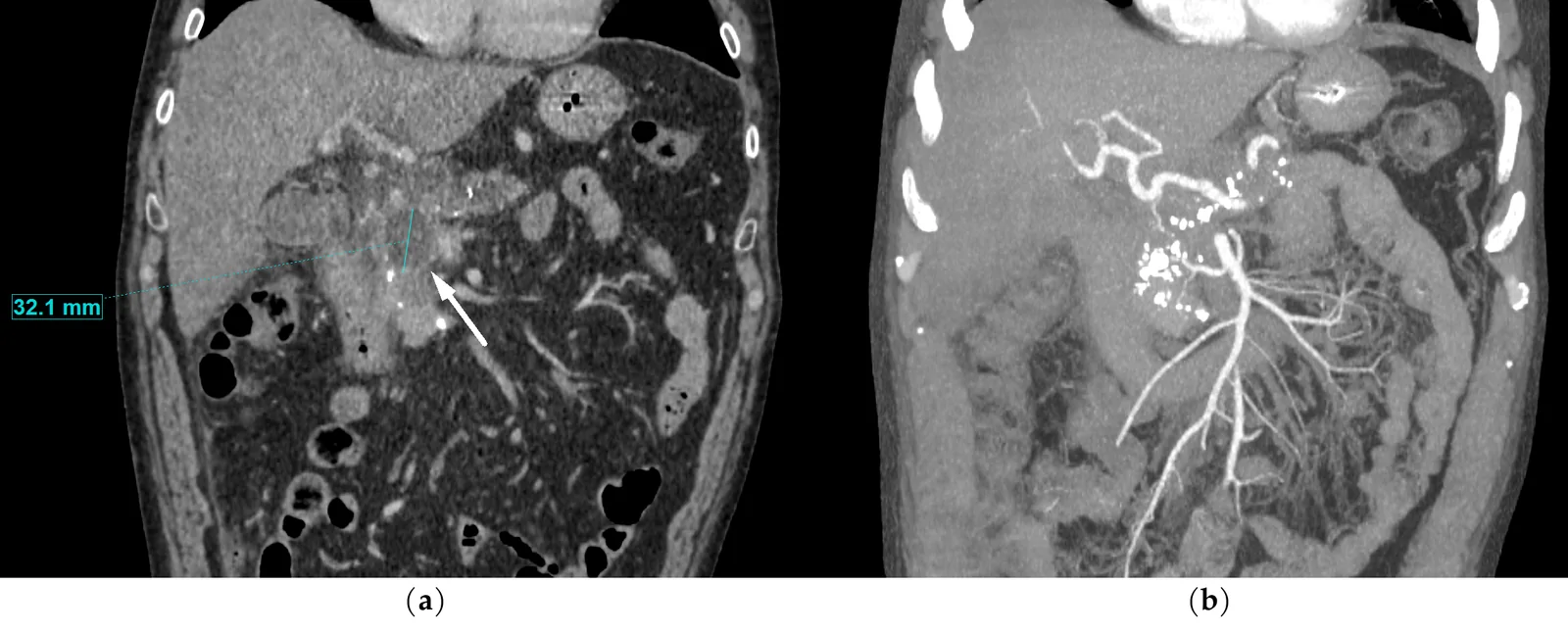
Arterial-phase images of the initial contrast-enhanced CT examination: coronal reformation (**a**) and corresponding coronal maximum intensity projection (MIP) (**b**). The largest acute necrotic collection is seen in the region of the pancreatic head, measuring approximately 32 mm (white arrow) (**a**). No pseudoaneurysm is identified on the arterial-phase images at this stage (**b**), indicating that the vascular complication developed later, during follow-up.

**Figure 3:**
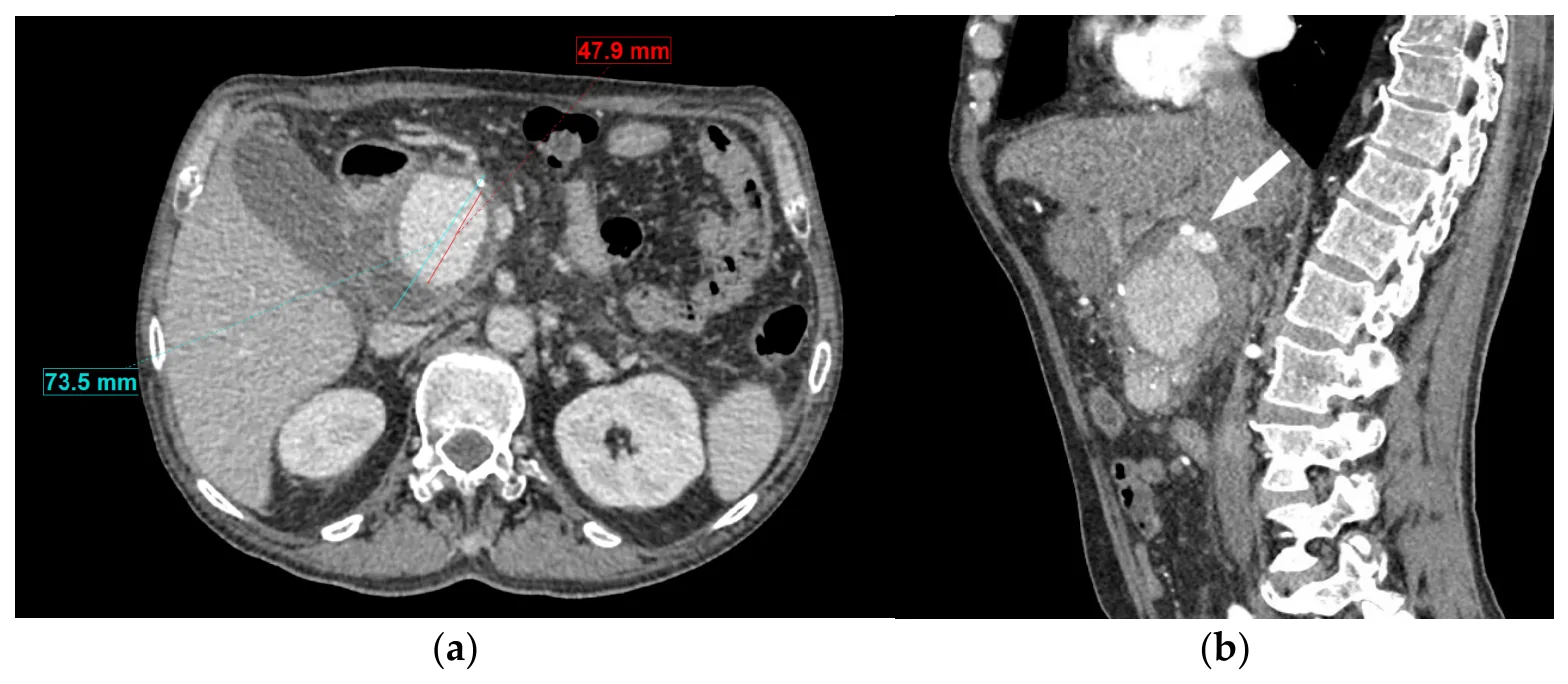
Axial and sagittal arterial-phase contrast-enhanced CT images obtained at the three-month post-discharge follow-up show a large, well-organized walled-off necrotic collection (WON) measuring approximately 73 mm in its greatest dimension and predominantly involving the pancreatic head and neck (cyan measurement line) (**a**). The collection has heterogeneous contents, with areas of fluid attenuation and necrotic debris, consistent with a late sequela of necrotizing pancreatitis. In the dependent portion of the necrotic collection, a well-circumscribed, round hyperattenuating lesion measuring approximately 48 mm (red measurement line in (**a**)) is identified (white arrow) (**b**). It shows homogeneous arterial-phase enhancement comparable to that of adjacent arteries, which is highly suggestive of a visceral artery pseudoaneurysm. On these non-selective images, the lesion is located in the region of the pancreatic head and neck, cannot be delineated as a separate entity and shows homogeneous enhancement without a jet phenomenon, suggesting a possible origin from the superior pancreaticoduodenal arcade; its origin from the proper hepatic artery was subsequently established by CT angiographic reconstruction and selective DSA (Figure 4 and Figure 5). No active contrast extravasation is seen. The remaining pancreatic parenchyma appears atrophic, with multiple parenchymal calcifications consistent with chronic pancreatitis. Multiple reactive subcentimeter peripancreatic lymph nodes are also seen.

**Figure 4:**
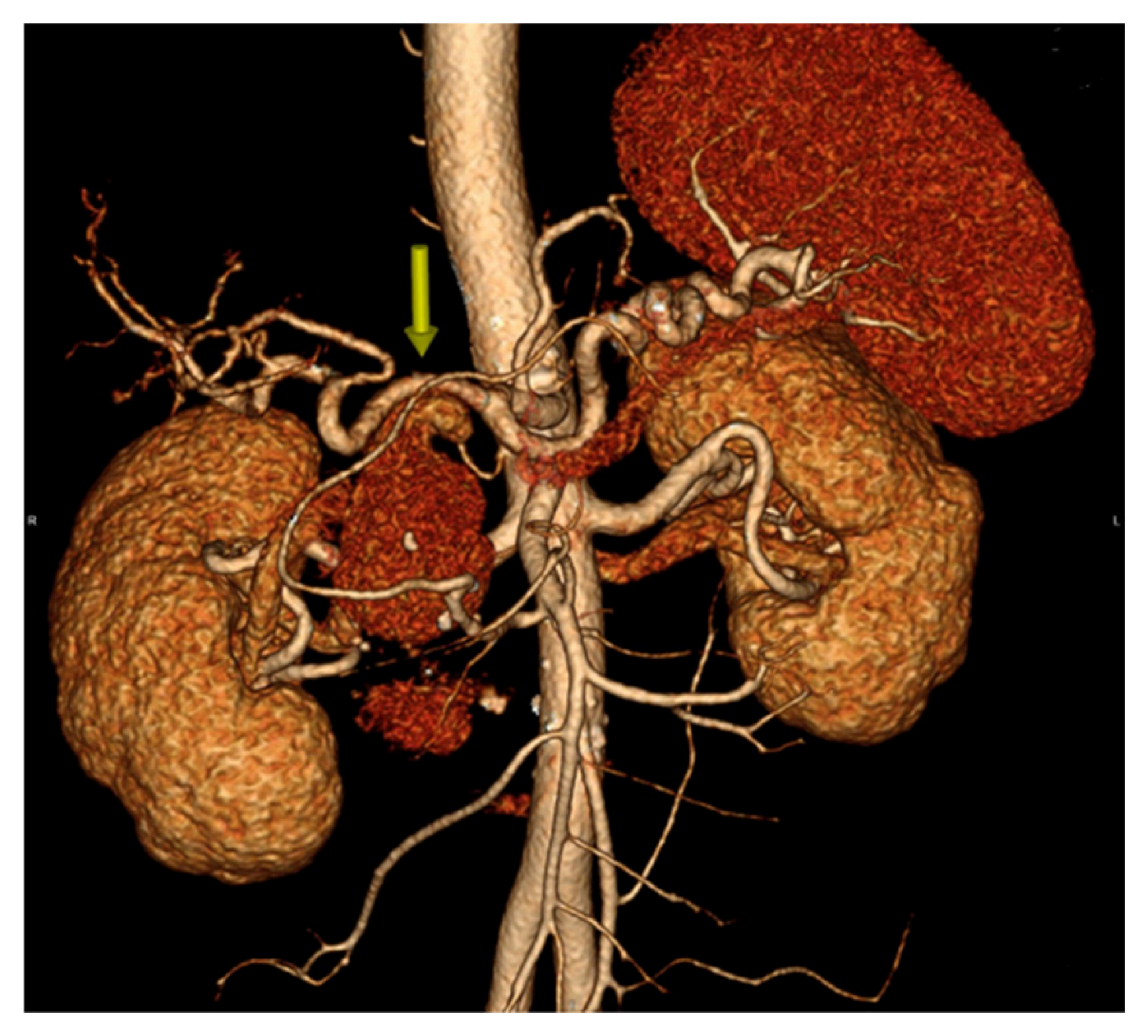
Three-dimensional volume-rendered CT angiography shows the pseudoaneurysm (yellow arrow) within the walled-off necrotic collection. Enhancement of the pseudoaneurysm is most pronounced in its upper part, adjacent to the proper hepatic artery, suggesting that it arises from this vessel; this could be confirmed only by selective catheter angiography (Figure 5), which guided further management.

**Figure 5:**
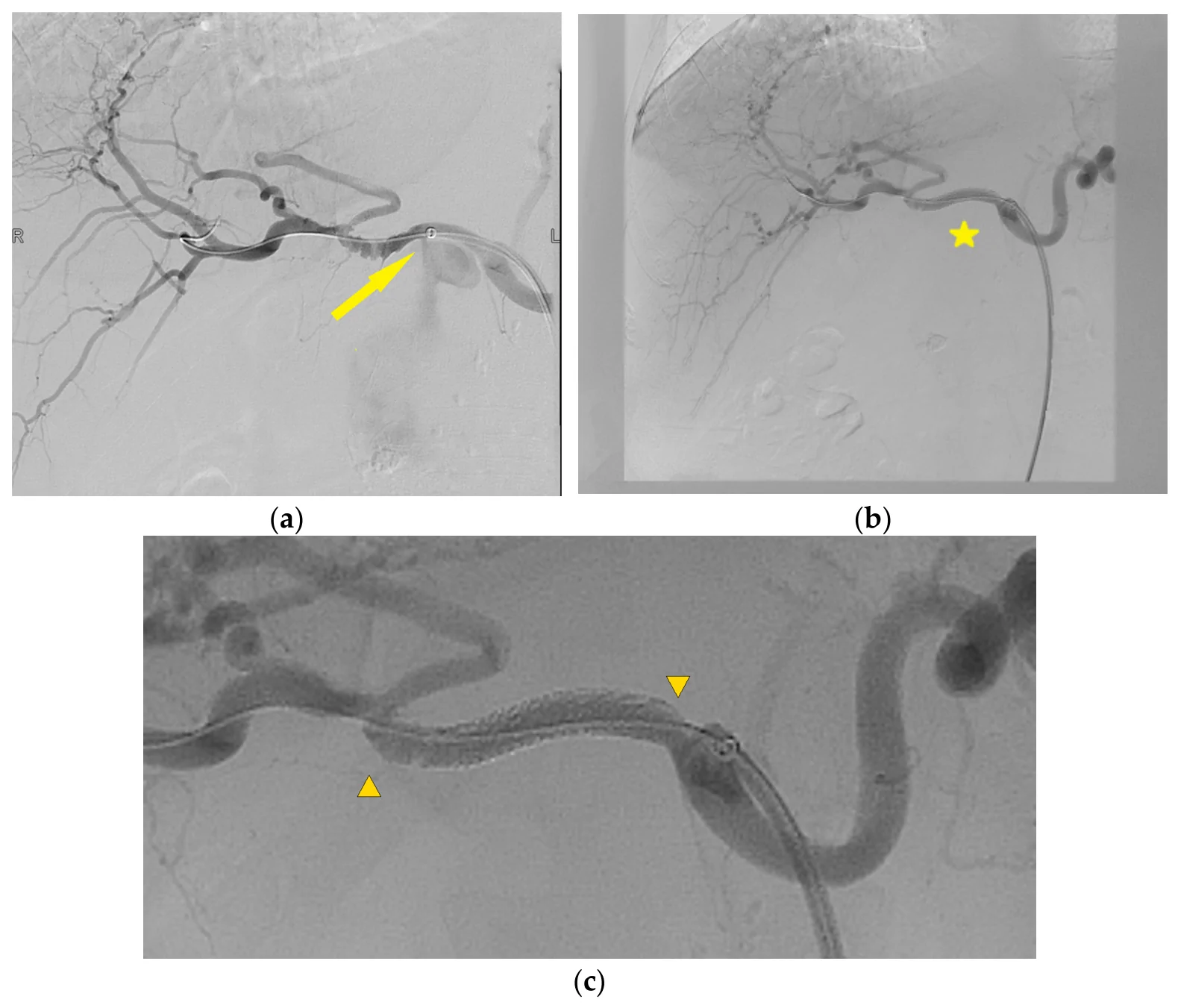
Digital subtraction angiography (DSA) performed in conjunction with planned covered stent implantation. Selective catheterization of the common hepatic artery via a right transfemoral approach confirmed the presence and exact location of the pseudoaneurysm arising from the proper hepatic artery (yellow arrow) (**a**), consistent with the prior CT angiographic findings. After successful guidewire passage through the affected arterial segment, a 6 × 38 mm covered stent (yellow asterisk) was deployed in the proper hepatic artery across the pseudoaneurysm neck (**b**). Post-deployment angiography demonstrated immediate and complete exclusion of the pseudoaneurysm sac, with no residual opacification or contrast extravasation. Antegrade flow through the stent lumen into the distal hepatic arterial branches was preserved, confirming maintained hepatic arterial perfusion. A magnified post-deployment view shows the covered stent within the proper hepatic artery, with both ends of the stent marked by yellow arrowheads (**c**).

**Figure 6:**
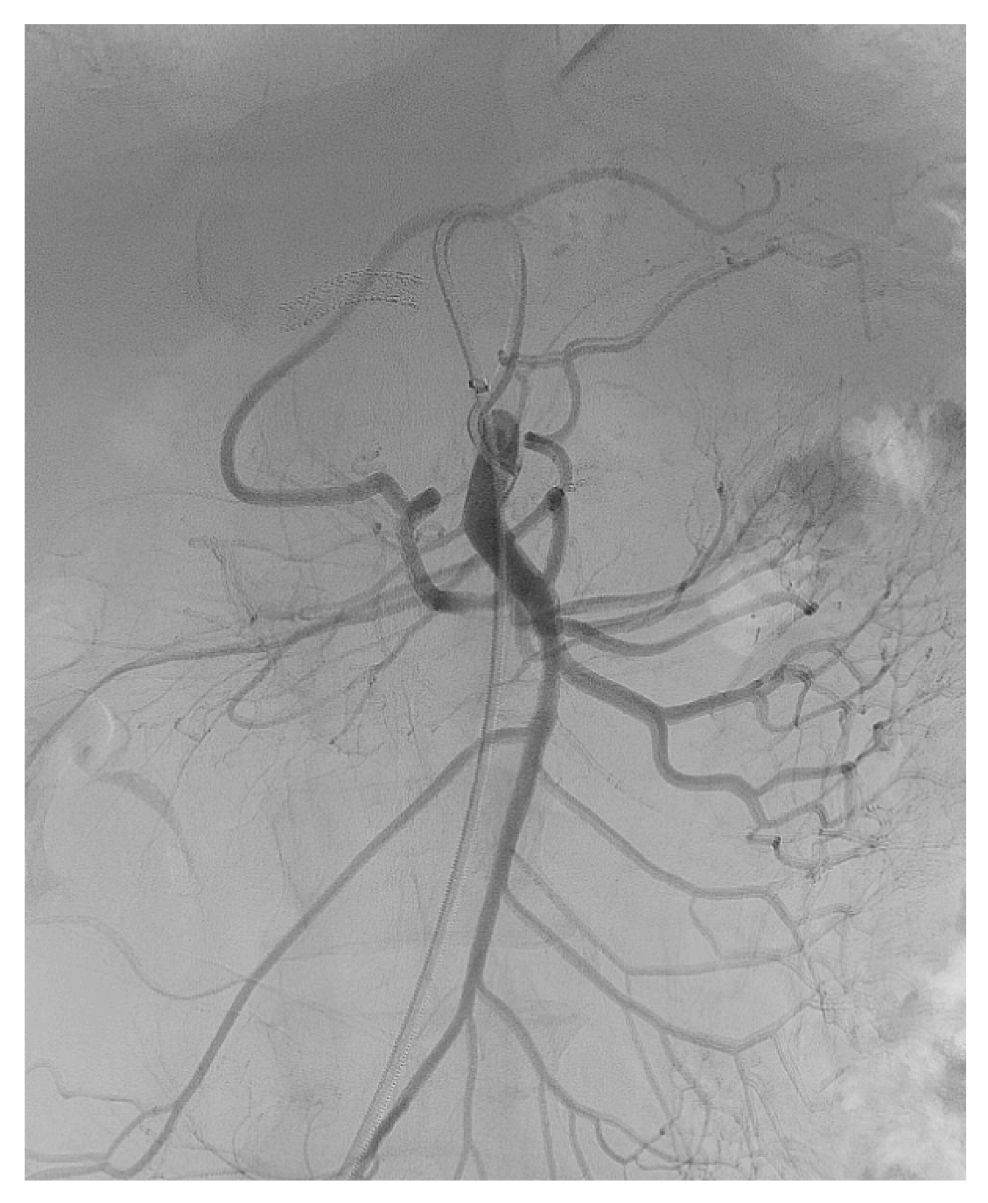
Selective angiography of the superior mesenteric artery was performed to exclude retrograde perfusion of the treated pseudoaneurysm and to look for additional visceral artery pseudoaneurysms. There was no retrograde filling of the excluded pseudoaneurysm sac via collateral pathways, including the pancreaticoduodenal arcade. No additional pseudoaneurysms were identified within the superior mesenteric arterial territory. These findings confirmed the absence of collateral reperfusion of the treated pseudoaneurysm. Covered stent placement thus achieved immediate exclusion of the pseudoaneurysm while preserving antegrade hepatic arterial flow, making it an effective, organ-preserving endovascular treatment strategy.

**Figure 7:**
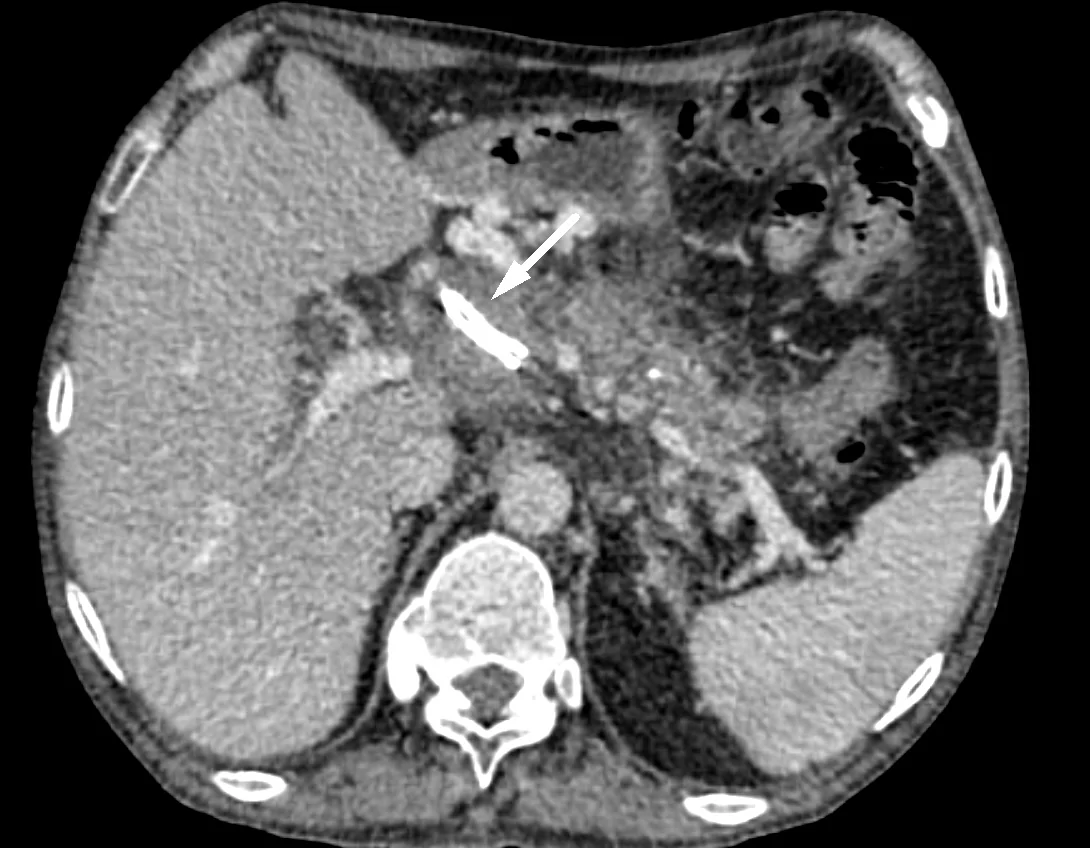
Follow-up contrast-enhanced CT performed three months after the procedure shows the covered stent in the proper hepatic artery (white arrow) with preserved luminal opacification, no contrast filling of the pseudoaneurysm sac and a reduction in the size of the necrotic collection.

## Data Availability

The data presented in this study are available from the corresponding author upon reasonable request.
